# Kaposi Sarcoma of Lymph Nodes

**DOI:** 10.1038/bjc.1970.55

**Published:** 1970-09

**Authors:** D. Bhana, A. C. Templeton, S. P. Master, S. K. Kyalwazi

## Abstract

**Images:**


### KAPOSI SARCOMA OF LYMPH NODES

D. BHANA, A. C. TEMPLETON, S. P. MASTER AND S. K. KYALWAZI

*From the Mulago Hospital and Makerere Medical School, Kampala, Uganda*

Received for publication May 1, 1970

**SUMMARY.—Sixteen out of 48 adult African patients with Kaposi sarcoma were found to have tumour tissue in lymph nodes. The evidence suggests that there are probably two main types of involvement. One occurs predominantly in younger patients and involves many groups of glands, probably develops *in situ*, and is associated with a poor prognosis. The other form is the result of metastasis to a node from an aggressive tumour in the neighbourhood. This occurs more commonly in the older patient and carries a much better prognosis than in those with generalised lymphadenopathy, though worse than in patients with nodular disease without gland involvement. Follow-up over a period of many years will be required to discover the outcome in these cases. Kaposi sarcoma is unusual in women but when it occurs runs a more aggressive course than in men.**

NEARLY a hundred years after its first description the aetiology and cell of origin of Kaposi sarcoma remains unknown. Initially regarded as a rare tumour affecting males of Central European and Mediterranean origin the disease is now known to have its highest incidence in Africa, in particular the tropical belt extending from the Congo across East Africa (Dupont *et al.*, 1948; Kaminer and Murray, 1950). In Uganda during the five-year period 1964–69, 359 cases were diagnosed histologically, that is 5·2% of all malignant tumours.

Although Kaposi himself described visceral involvement in one case coming to autopsy (Rothman, 1962), it is surprising that for a considerable period the disease was regarded as a purely dermatological condition. Increasing experience (Cox and Hellwig, 1959; Lothe and Murray, 1962) has shown that this is a systemic disease of multicentric origin and that the cutaneous manifestations are frequently associated with visceral lesions which are clinically quiescent. Occasionally the tumour may arise in one of the viscera without evident dermal disease.

An interesting form of this tumour occurs in children in whom the lymph nodes are mainly affected; this form pursues a rapid fulminant course and is usually resistant to treatment (Davies and Lothe, 1962). Primary Kaposi sarcoma has also been reported in the adult as an uncommon presentation (Ecklund and Valaitis, 1962; Lee and Moore, 1965). The incidence of lymph node involvement in adult patients, however, remains unknown.

In the present study an attempt was made to study the frequency of lymph node involvement in 48 adult patients with Kaposi sarcoma and to see if there was any correlation between lymph node pattern and the clinical behaviour of the disease.

### MATERIALS AND METHODS

Forty-eight adult patients with histologically proved Kaposi sarcoma admitted to Mulago Hospital, Kampala, Uganda, between April 1968 and January 1970

were studied. Full clinical evaluation was supplemented by radiological examination of chest, soft tissues, and bone for evidence of tumour. A proportion of patients had lymphangiography to detect lymph node involvement. Of the 48 patients 44 were male and 4 female. The age range at onset was from 18 to 70 years (mean 37 years) (Fig. 1). All patients had a biopsy of tumour to confirm diagnosis and a further gland biopsy was performed on 44 patients. A gland from the vertical and horizontal chain of inguinal glands was removed from each groin. For technical reasons inguinal biopsy was done where the disease was widespread and involved the upper limbs as well. All material was serially sectioned into 4 mm. slices which were then processed and histological examination was carried out on all slices. Slides were stained with haematoxylin and eosin, a reticulin stain and methyl green pyronin.

Fig. 1.—Shows age at onset with percentage of total in each decade.

### RESULTS

Two patients presented with lymphadenopathy alone without dermal involvement. In the remaining 46 patients there was cutaneous disease and in all but one the lower limbs were affected. 16 out of 48 patients ($33\frac{1}{3}\%$) showed tumour in lymph nodes. Two points were noteworthy. All 4 female patients had involvement of nodes. Involved nodes were frequently only moderately enlarged and could, on naked eye examination alone, be passed as normal while large, fleshy glands were almost invariably free of tumour.

The main clinical features of the 16 patients with glandular disease are summarised in Table I. It was found that the patients were divisible into four groups on the basis of clinical presentation.

TABLE I

| Group | Case No. | Age at onset | Sex | Extent of disease | Duration | Present status |
|---|---|---|---|---|---|---|
| A | 1 | 30 | M | Kaposi sarcoma glands in (R) groin recurred after surgical excision 3 times | 10 years | Died of bilateral chest infection |
| A | 2 | 23 | M | Generalised lymphadenopathy, bilateral pleural effusion, ascites, generalised oedema | 8 months | Died of disease |
| B | 3 | 20 | M | Generalised lymphadenopathy, oedema, widespread florid cutaneous lesions of limbs and trunks | 3 years | Died of disease |
| B | 4 | 22 | M | Generalised lymphadenopathy, widespread cutaneous nodules all limbs | 2 years | Died of disease |
| B | 5 | 27 | F | Generalised lymphadenopathy, tumour in pharynx, rectum, oedema limbs | 15 months | Alive but with disease |
| B | 6 | 29 | F | Generalised lymphadenopathy, widespread nodules on body surface | 9 months | Died of disease |
| B | 7 | 55 | M | Generalised lymphadenopathy, nodules in limbs, plaques in (R) thigh | 3 months | Took own discharge |
| C | 8 | 24 | M | Nodular Kaposi left leg, oedema, groin glands involved | 1 year | Alive and well |
| C | 9 | 28 | M | Multiple fungating tumours. Reccurence at and proximal to stump following below-knee amputation 2 years ago | 8 years | Alive, disease under control |
| C | 10 | 36 | M | Kaposi nodules (R) leg-fungation of glands in groin | 1 year | Died of disease |
| C | 11 | 39 | M | Nodular disease both legs, oedema both legs | 1 year | Alive and well |
| C | 12 | 39 | F | Nodular disease (L) leg + (R) forearm, oedema leg | 2 years | Alive and well |
| C | 13 | 54 | M | Nodules on dorsum of left foot, ulcerative lesion on lateral aspect of foot | 18 months | Alive and well |
| C | 14 | 58 | F | Mixed infiltrative and florid nodules both lower limbs, oedema | 2 years | Died of drug toxicity |
| C | 15 | 68 | M | Fungating lesion sole of foot, nodules dorsum left hand | 2 years | Alive and well |
| D | 16 | 23 | M | One submental gland only. 2 nodules on left upper limb | 18 months | Alive and well |

*Group A. Primary tumour of lymph glands without cutaneous disease*

There were two patients in this group (Cases 1 and 2).

*Case* 1.—The first patient was a 40 year old Ruanda man who gave a history of a mass growing in the right groin for 10 years. This had been excised three times but had always recurred. He was admitted to Mulago Hospital on September 21st, 1968 with a soft fluctuant swelling in the right iliac fossa which was thought to be due to involvement of iliac nodes.

Biopsy showed replacement of node by Kaposi sarcoma. He was given two courses of Actinomycin D and the tumour began to show signs of regression, but about a week later he developed bilateral bronchopneumonia and died. At necropsy he was shown to have bilateral lung abscesses but there was no evidence of Kaposi sarcoma except in the right groin.

*Case* 2.—A 23 year old Etesot male was referred to Mulago Hospital on August 20th, 1968. He had been well until 8 months previously when he developed swelling of the left leg with some pain. Shortly after this he had developed gradual distension of his abdomen and enlargement of glands in the neck, axillae and groins. On admission, his general condition was very poor. He had generalised lymph node enlargement and evidence of bilateral pleural effusion and ascites. Biopsy of a cervical lymph node showed Kaposi sarcoma. The patient's general condition deteriorated rapidly and he died on September 1st, 1968.

Postmortem examination showed bilateral haemorrhagic pleural effusion and haemorrhagic ascites with widespread Kaposi sarcoma of small and large intestine, kidney, spleen, retroperitoneal lymph nodes.

*Group B. Generalised lymphadenopathy with widespread cutaneous disease*

(Cases 3, 4, 5, 6, 7.)

Three males and 2 females. Four out of the 5 patients were under the age of 30 and the disease pursued a downhill course although less rapidly than in the former group.

The general features of this group are illustrated by the history of Case 3.

A 22 year old Muganda farmer was admitted to hospital on January 2nd, 1970. About 3 years before he had begun to develop swelling of the lower limbs followed some months later by swelling of the upper limbs. He had received treatment for these at a dispensary near his home but without relief. About a year later he had developed multiple nodules of both feet which had increased in size and spread to involve the trunk and both upper limbs. During the 9 months or so before admission he had had several episodes of haemoptysis. On examination he had generalised oedema including the face. There were florid tumour nodules on all the limbs, the trunk and scrotum and there was gross abdominal distension with ascites. There was also generalised lymphadenopathy. He failed to respond to treatment and died on January 9th 1970.

Necropsy showed Kaposi tumour in the tonsil, kidneys, spleen, lymph nodes, pancreas, small bowel and bones including the vertebral column. Haemoptysis was a result of erosion of the bronchus by a neighbouring lymph node containing tumour.

*Group C. Involvement of a single group of glands draining an area of dermal involvement*

There were 8 patients in this group (Cases 8–15). All had deposit in glands draining the tumour-bearing area while the contralateral glands were unaffected. Patients in this group tended to be older, only 2 of the cases being under 30 years of age. The cutaneous lesions tended to be larger than usual and were frequently fungating or ulcerated. These lesions were sometimes solitary but were more often surrounded by multiple skin nodules.

Case 15 is typical of this group. A 70 year old Nubian male developed small nodules on the dorsum of his left hand 2 years ago. About the same time he noticed a painful nodule on the sole of his right foot. The nodules on the hand remained static but that on the sole continued to grow and eventually fungated. Inguinal gland biopsy showed tumour in the vertical glands on the right side. He was treated with Actinomycin D with regression of tumour.

*Group D. Involvement of single group of glands without regional disease*

There was one patient in this group (Case 16).

A 23 year old Muganda male was seen in the surgical clinic with a slightly painful subcutaneous nodule under the chin of 8 months duration. He also had two pea-sized cutaneous nodules on the left upper limb. An excision biopsy of the submental nodule showed a lymph node replaced by Kaposi sarcoma. 15 months after treatment he has remained well and free of disease.

### DISCUSSION

Previous reports have described the occurrence of Kaposi sarcoma in lymph nodes as an uncommon node of presentation in adults (Ecklund and Valaitis, 1962; Lee and Moore, 1965). However, the incidence of gland involvement found at necropsy in patients dying of the disease is very much higher (Lothe and Murray, 1962). Cox and Hellwig (1959) found gland involvement in 10 out of 14 cases and was the most common site of extracutaneous disease.

The present study on living patients with Kaposi sarcoma has shown that $33\frac{1}{3}\%$ of cases had tumour in lymph nodes. The pattern of involvement varied widely but its extent and form appeared to bear a relationship to the clinical course of the disease. When an isolated group of glands was involved the tumour pursued a slow, indolent course, remaining confined for a considerable period. Whether this form of disease ever disseminates is an open question and can only be answered by a long period of follow-up. It is, however, reasonable to presume that with adequate treatment by surgery and/or radiotherapy it might be possible to eradicate the disease completely. Lee and Moore (1965) described a patient who was alive and well 15 years after excision biopsy and had not required any further treatment. One of our patients who died of intercurrent infection 10 years after initial diagnosis was found, at autopsy, to have disease confined only to the inguinal and esternal iliac group of glands on one side. The other (Case 16) is alive and well 15 months after onset of symptoms.

When there was generalised lymphadenopathy, however, the course was strikingly different. The disease appeared to grow with an explosive force, overwhelming the defence mechanism of the body, and, as in the childhood form, there was an indifferent response to treatment with a fatal outcome in a matter of months. Slavin *et al.* (1969) described one patent of 18 years with generalised lymphadenopathy, oedema of lower limbs and hepatosplenomegaly who died within 4 months of onset. In our series 4 of the 6 patients presenting with generalised lymphadenopathy have died within 3 years of the onset of disease, the fate of another case is unknown and the sixth still has active disease 15 months after onset which has shown repeated relapse after treatment. This presentation was seen most frequently in the younger age group, 5 of the 6 cases being under 30 years of age.

In the remaining 8 patients tumour was present only in glands draining an area of cutaneous disease while the contralateral glands were unaffected. It is possible that these cases represent true examples of metastases. The prognostic significance of this finding is not wholly clear but it has been shown (Kyalwazi, 1969) that the late mortality in Kaposi sarcoma follows the development of a fungating lesion and it may be that metastases to lymph nodes occurs in this group.

Glandular involvement appeard to occur early in the course of the disease (Fig. 2) and affected a higher percentage in the younger age group in a proportion of whom the disease ran a rapid fatal course. In older patients the pattern usually seen was involvement of glands draining a fungating or ulcerating lesion on a limb. In such patients there was a greater tendency to involve viscera or bone. The progression of disease, however, was slow and most cases showed a satisfactory response to therapy (Kyalwazi, 1969; Kyalwazi *et al.*, 1970).

The mortality from disease varied in the different age group. In the 22 patients under the age of 30 there were 5 who died of disease (22·7%) while of 26 patients over the age of 30 only one died of disease and one further patient died

FIG. 2.—Shows duration of disease at time of biopsy in relation to glandular pattern.

of drug toxicity. These findings lend support to the view that the prognosis was poor when the disease occurred in the young adult and especially when viscera and lymph glands were involved (Cox and Hellwig, 1959).

Histologically the nodules of tumour frequently occurred in the periphery of the lymph node (Fig. 3), just as with metastases from carcinomas, in those with gland involvement regional to disease. This pattern was not seen in cases with generalised lymph node involvement; in these the tumour appeared to develop in the medulla of the node and gradually expand the node from within (Fig. 4). In those with disease in isolated glands the type of involvement was not clear as the nodes were completely replaced by tumour but it is probable that tumour had developed from the medulla.

The disease was much less common in women (4 females : 44 males in this series) but in all cases the disease was of an aggressive type. Two patients have died, one had widespread active disease despite treatment and only one is alive and well. It appeared that the more indolent nodular form of Kaposi was a rarity in females and the prognosis was significantly worse than in men.

We would like to record our thanks to the following: The Chief Medical Officer, Ministry of Health, Uganda, for permission to publish. The Medical Illustration Department, Makerere University College, for the illustrations. Mrs. Z. Pereira for secretarial help.

### REFERENCES

Cox, F. H. and Helwig, E. B.—(1959) *Cancer, N.Y.*, **12**, 289.
Davies, J. N. P. and Lothe, F.—(1962) *Acta Un. int. Cancr.*, **18**, 394.
Dupont, A., Chabeuf and Vanbreuseghern.—(1948) *Archs belg. Derm., Syph.*, **4**, 132.
Ecklund, R. E. and Valaitis, J.—(1962) *Archs Path.*, **74**, 224.
Kaminer, B. and Murray, J. F.—(1950) *S. Afr. J. clin. Sci.*, **1**, 1–25.
Kyalwazi, S. K. (1969)—*E. Afr. med. J.*, **46**, 8.
Kyalwazi, S. K., Bhana, D. and Master, S. P.—(1970) *E. Afr. med. J.* (In Press.)
Lee, S. C. H. and Moore, O. S.—(1965) *Archs Path.*, **80**, 651.
Lothe, F. and Murray, J. F.—(1962) *Acta Un. int. Cancr.*, **18**, 413.
Rothman, S.—(1962) *Acta Un. int. Cancr.*, **18**, 322.
Slavin, G., Cameron, H. Mc. D. and Singh, H.—(1969) *Br. J. Cancer*, **13**, 349.

---

### EXPLANATION OF PLATE

Fig. 3.—Shows nodules of tumour in periphery of lymph node.
Fig. 4.—Shows tumour expanding lymph node from the centre.

3

4

Bhana, Templeton, Master and Kyalwazi


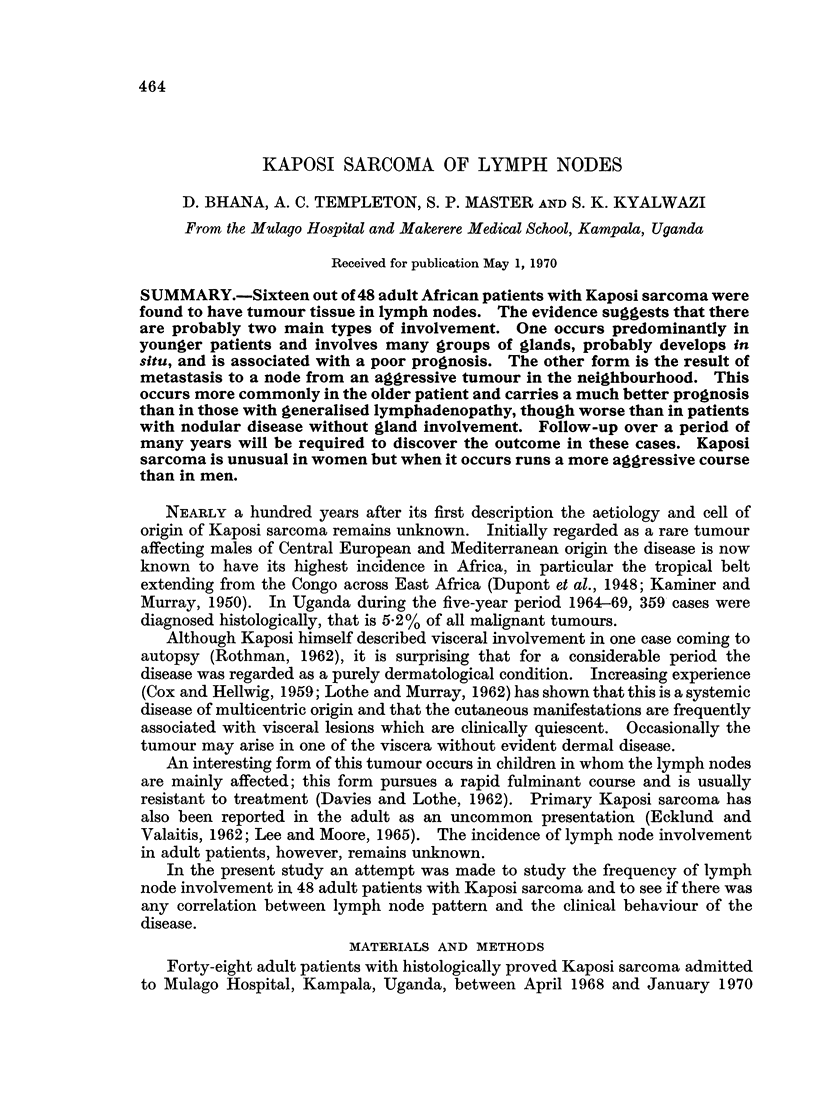

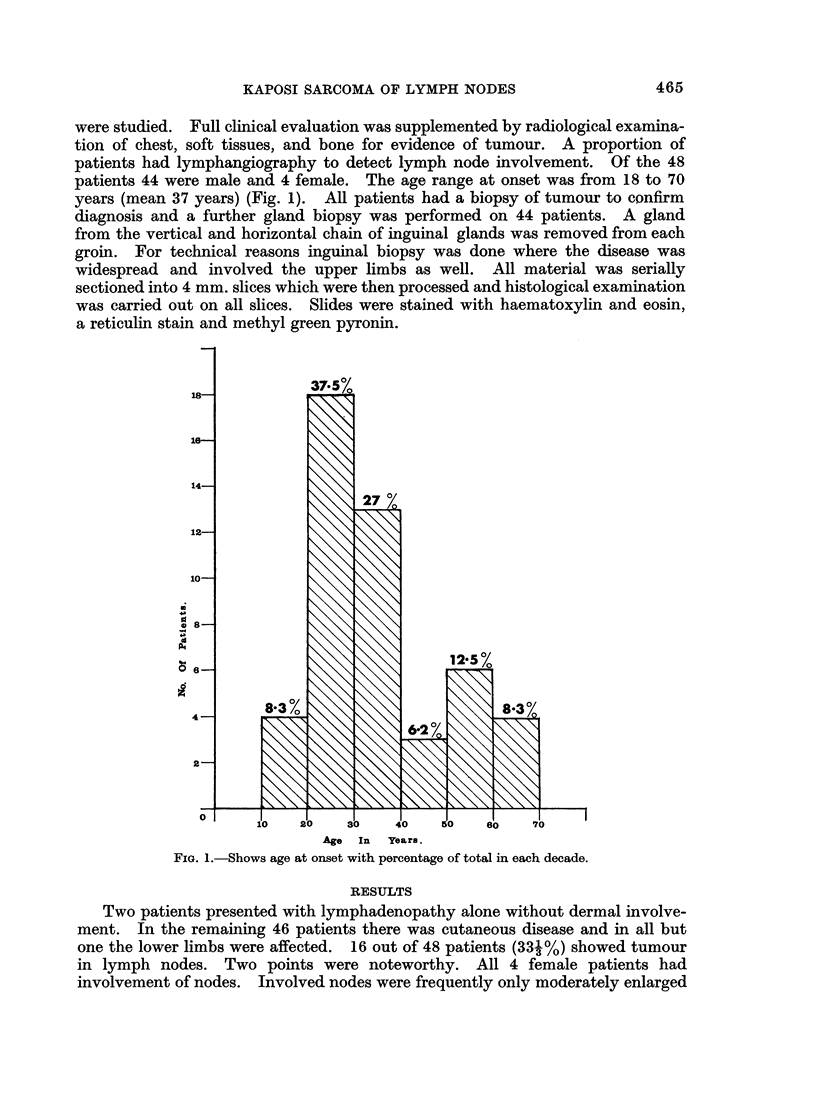

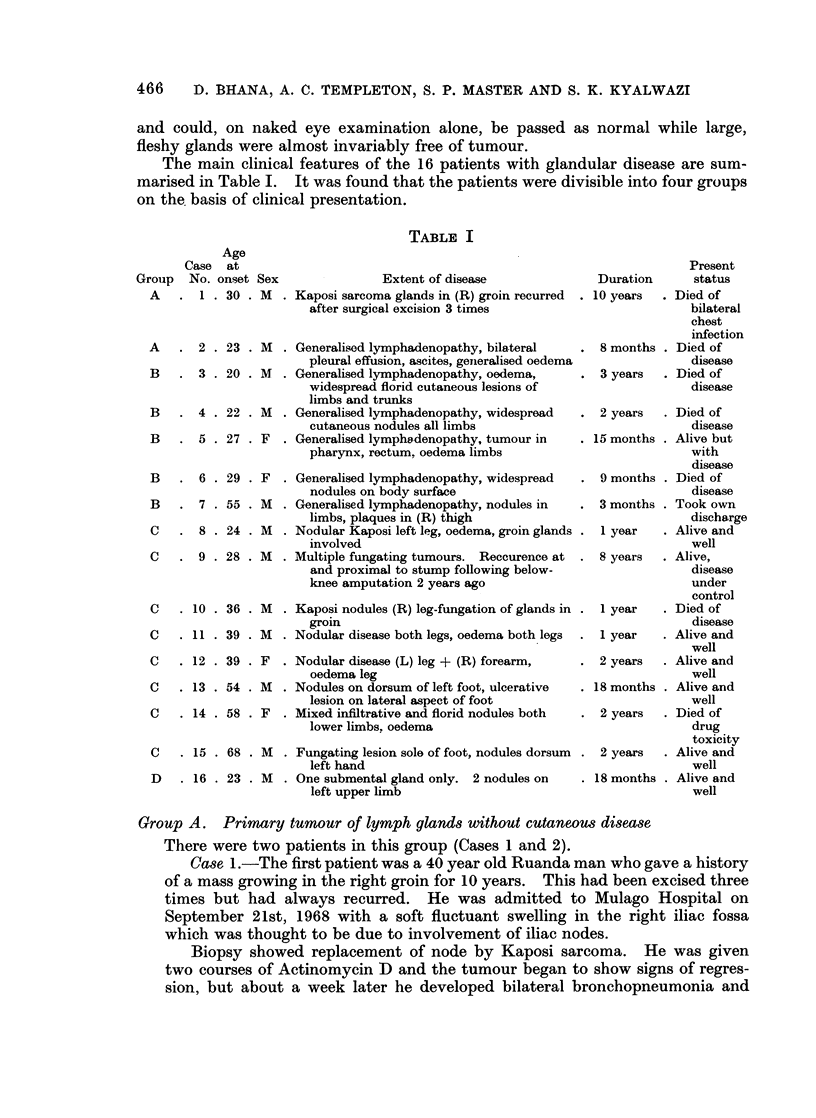

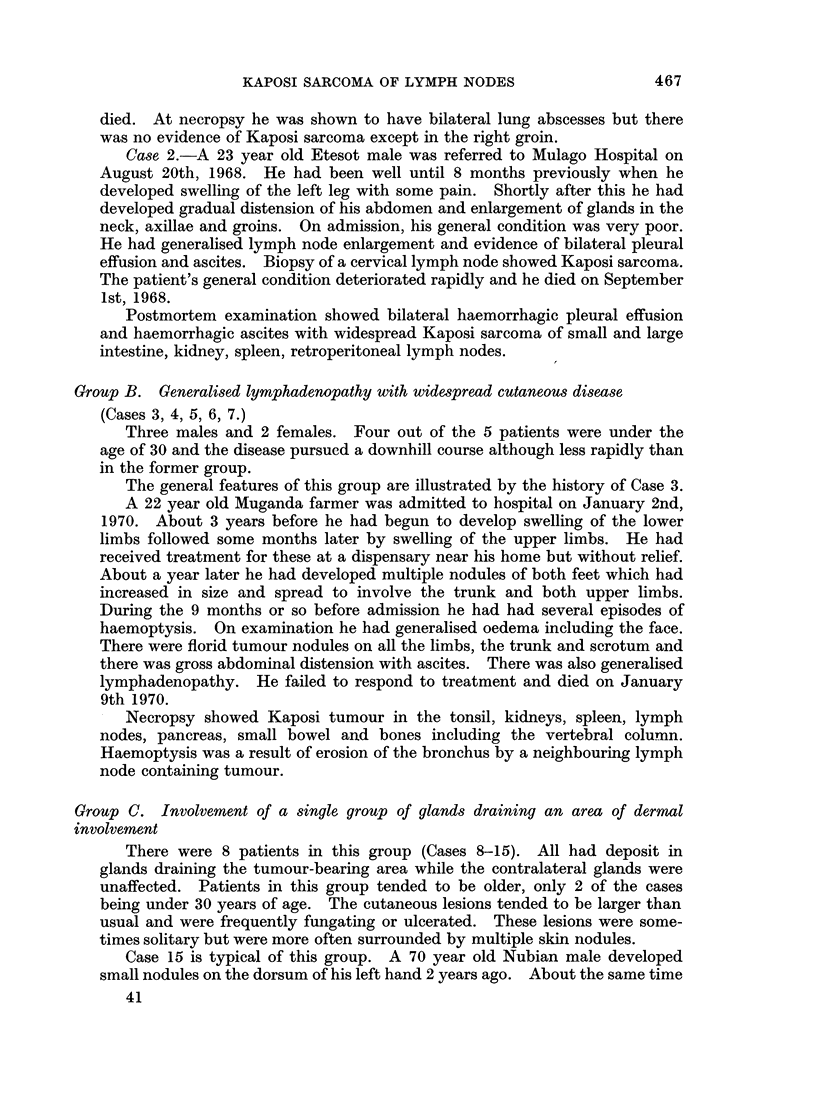

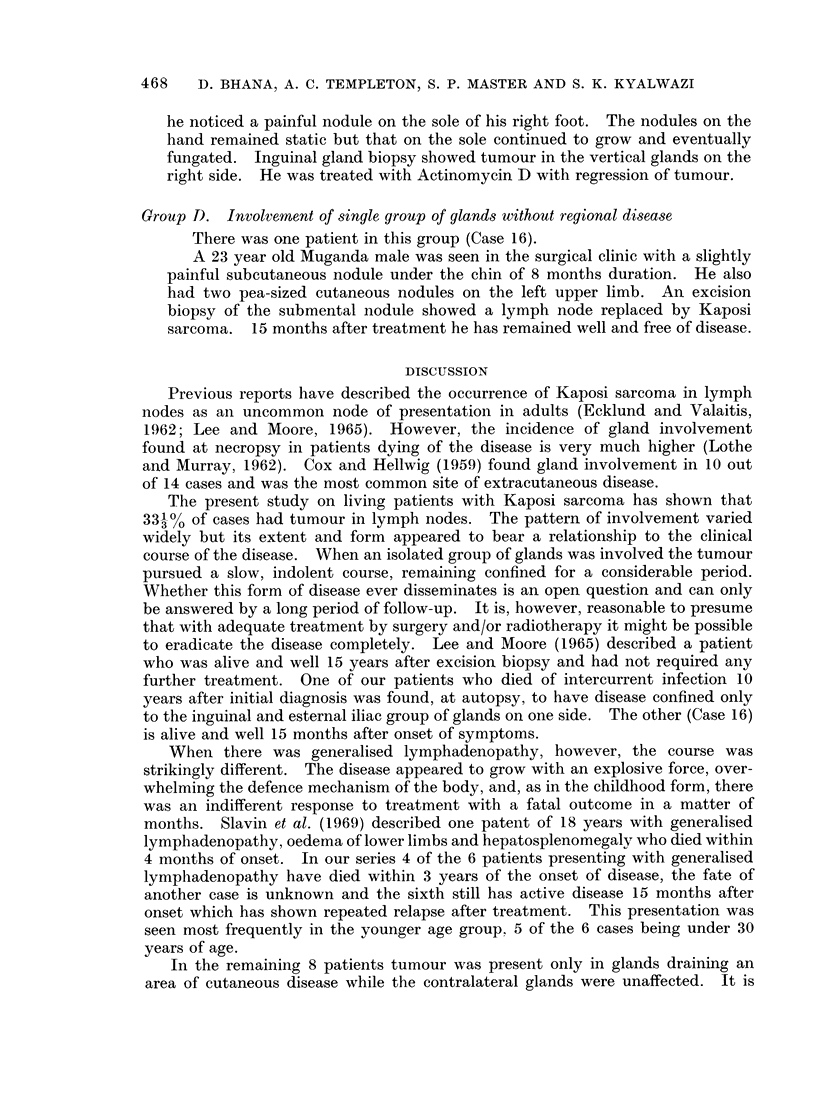

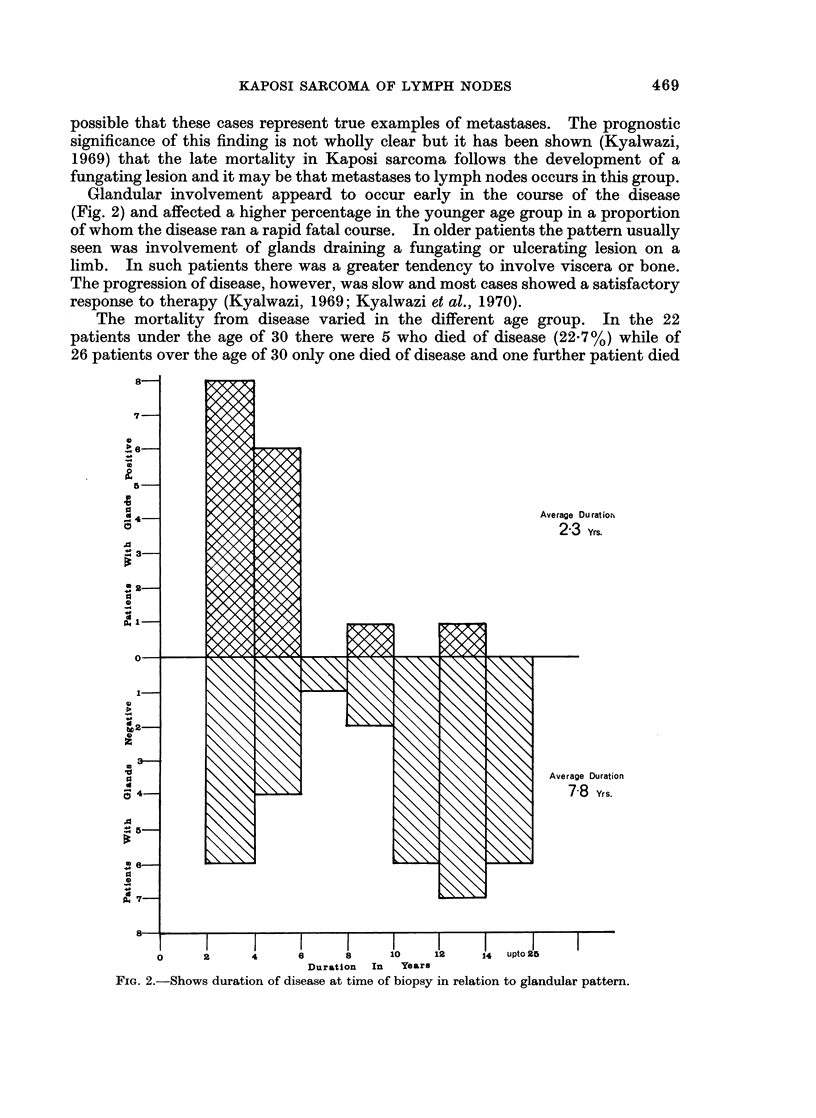

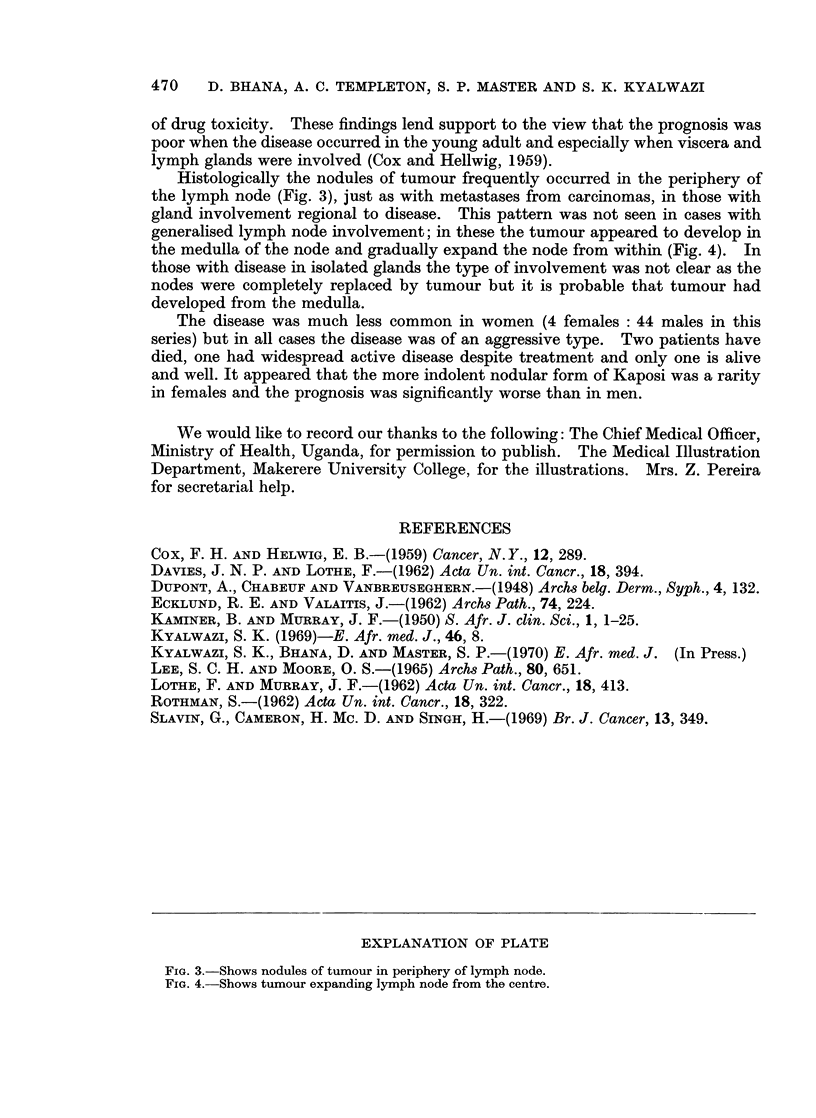

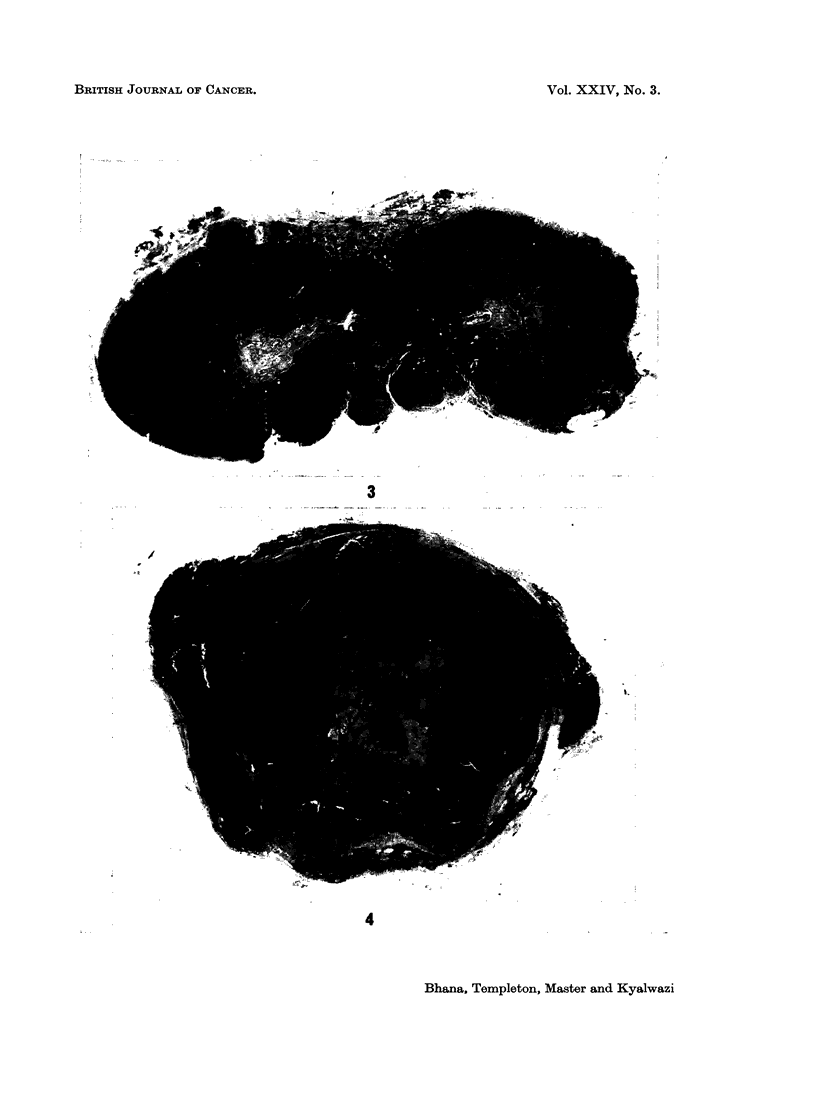


## References

[OCR_00626] COX F. H., HELWIG E. B. (1959). Kaposi's sarcoma.. Cancer.

[OCR_00628] DAVIES J. N., LOTHE F. (1962). Kaposi's sarcoma in African children.. Acta Unio Int Contra Cancrum.

[OCR_00633] KAMINER B., MURRAY J. F. (1950). Sarcoma idiopathicum multiplex haemorrhagicum of Kaposi, with special reference to its incidence in the South African Negro, and two case reports.. S Afr J Clin Sci.

[OCR_00640] ROTHMAN S. (1962). Some remarks on Moricz KAPOSI and the history of Kaposi's sarcoma.. Acta Unio Int Contra Cancrum.

[OCR_00642] Slavin G., Cameron H. M., Singh H. (1969). Kaposi's sarcoma in mainland Tanzania: a report of 117 cases.. Br J Cancer.

